# Profiling of circulating exosomal miRNAs in patients with Waldenström Macroglobulinemia

**DOI:** 10.1371/journal.pone.0204589

**Published:** 2018-10-04

**Authors:** Juliette M. Bouyssou, Chia-Jen Liu, Mark Bustoros, Romanos Sklavenitis-Pistofidis, Yosra Aljawai, Salomon Manier, Amir Yosef, Antonio Sacco, Katsutoshi Kokubun, Shokichi Tsukamoto, Adriana Perilla Glen, Daisy Huynh, Jorge J. Castillo, Steven P. Treon, Véronique Leblond, Olivier Hermine, Aldo M. Roccaro, Irene M. Ghobrial, Marzia Capelletti

**Affiliations:** 1 Department of Medical Oncology, Dana-Farber Cancer Institute, Harvard Medical School, Boston MA, United States of America; 2 Université Paris-Saclay / Hôpital Necker-Enfants Malades, Paris, France; 3 Division of Hematology and Oncology, Department of Medicine, Taipei Veterans General Hospital, Taipei, Taiwan; 4 School of Medicine, National Yang-Ming University, Taipei, Taiwan; 5 Department of Hematology at Pitié Salpêtrière Hospital, Paris, France; 6 INSERM UMR 1163, Laboratory of Cellular and Molecular Mechanisms of Hematological Disorders and Therapeutic Implications, Paris, France; Gustave Roussy, FRANCE

## Abstract

Waldenström Macroglobulinemia (WM) is a low-grade B-cell lymphoma characterized by disease progression from IgM MGUS to asymptomatic and then symptomatic disease states. We profiled exosomes from the peripheral blood of patients with WM at different stages (30 smoldering/asymptomatic WM, 44 symptomatic WM samples and 10 healthy controls) to define their role as potential biomarkers of disease progression. In this study, we showed that circulating exosomes and their miRNA content represent unique markers of the tumor and its microenvironment. We observed similar levels of miRNAs in exosomes from patients with asymptomatic (smoldering) and symptomatic WM, suggesting that environmental and clonal changes occur in patients at early stages of disease progression before symptoms occur. Moreover, we identified a small group of miRNAs whose expression correlated directly or inversely with the disease status of patients, notably the known tumor suppressor miRNAs let-7d and the oncogene miR-21 as well as miR-192 and miR-320b. The study of these miRNAs’ specific effect in WM cells could help us gain further insights on the mechanisms underlying WM pathogenesis and reveal their potential as novel therapeutic targets for this disease.

## Introduction

Waldenström Macroglobulinemia (WM) is a low-grade B-cell lymphoma that is characterized mainly by infiltration of the bone marrow with lymphoplasmacytic cells and the secretion of an immunoglobulin M (IgM) protein[1]. There are distinct stages of disease progression for WM, starting from the precursor condition termed IgM monoclonal gammopathy of undetermined significance (IgM MGUS) that is defined by a bone marrow infiltration below10%. IgM MGUS has a rate of progression of 1.5–2% risk of progression to WM per year[2]. Patients with smoldering/asymptomatic WM display no major symptoms and are observed until their disease progresses to symptomatic WM[3]. The later disease state is characterized by symptoms such as anemia, peripheral neuropathy or organomegaly[3]. Despite the differences in clinical presentation of these stages of the disease, the molecular mechanisms underlying WM progression largely remain to be elucidated[4–6].

Exosome are 50–140 nm vesicles secreted by cells of different types including tumor cells[7]. They mediate intercellular communication by transporting a cargo of lipids, proteins and nucleic acids and transferring it to recipient cells[7]. It has been shown that exosomes can promote cancer progression through the regulation of various oncogenic processes including reprogramming of the tumor microenvironment, metastasis, immune escape and drug resistance[8, 9]. Interestingly, these oncogenic effects can be carried out by the miRNAs contained in these exosomes. In fact, different studies have shown that exosomal miRNAs can have prognostic and biological implications in the pathogenesis of many cancers[10–14].

MicroRNAs (miRNAs) are small, non-coding 19–22 nucleotide long RNAs that exert post-transcriptional regulation by binding the 3’UTR region and in some cases the coding region of target messenger RNAs, hence inhibiting their translation[15, 16]. Due to their ability to modulate essential cellular processes, miRNAs play an important role in tumorigenesis and their deregulation is a hallmark of cancer[17, 18]. In fact, large numbers of miRNAs have demonstrated oncogenic or tumor suppressor properties in specific cellular contexts[19, 20].

There is evidence that miRNAs are not randomly sorted but rather actively incorporated into exosomes[21]. Furthermore, it has been reported that precursor miRNAs can be processed into mature miRNAs inside tumor-derived exosomes carrying the microRNA biogenesis machinery[13]. This supports the notion that exosomal miRNAs can play an active role in recipient cells.

Exosomes can travel through body fluids to transfer their cargo and can be isolated from peripheral blood[21]. Circulating exosomes originate from various cell types including tumor cells, immune cells and cells of the tumor microenvironment. The analysis of these exosomes’content can provide valuable information on molecular changes occurring during disease progression. A number of recent studies have identified exosomal miRNAs with a promising potential for cancer diagnosis and prognosis, including one study in patients with multiple myeloma, a hematologic malignancy biologically close to WM[22–26]. Furthermore, miRNAs from circulating exosomes display a number of advantages as prognosis markers, including their high stability thanks to the protection from nucleases conferred by the exosomal membrane and the low invasiveness of the sampling method[26]. Therefore, we sought to examine the content of the circulating exosomes of WM patients to identify specific miRNAs that are deregulated during disease progression.

## Material and methods

### Patient samples

Peripheral blood and bone marrow biopsy samples were collected between 2007 and 2016 from patients with WM and healthy controls after approval from the Dana-Farber Cancer Institute Institutional Review Board (IRB# 09–233) with written informed consent. No minors were included in this research. Informed consent was obtained from all patients in accordance with the Declaration of Helsinki. The patients’ disease status was determined based on the Mayo stratification of Macroglobulinemia[2]. A total of 84 samples were analyzed including 10 healthy controls, 30 smoldering WM and 44 symptomatic WM. The mean age of the 10 healthy controls was 38 years old.

### Exosome isolation and characterization

We used an ultracentrifugation method to isolate exosomes from plasma (2 to 10 mL). The detailed isolation procedure is shown in S1A Fig. The expected exosomes size range was confirmed by electron microscopy using antibodies directed against CD63 and CD81 as previously described and by particle size analysis with a NanoSight NS300 Instrument (Malvern instruments)[7].

### Cell lines

The BCWM.1 and MWCL-1 cell lines were kindly gifted to us from Dr. Steven Treon (Dana Farber Cancer Institute, Harvard, MA) and Dr. Steven Ansell (Mayo Clinic, Rochester, MN). Cell lines were cultured in RPMI-1640 medium containing 10% fetal bovine serum, 2 μM l-glutamine, 100 U/mL penicillin, and 100 μg/mL streptomycin. Exosome-depleted culture medium was prepared by centrifuging the supplemented RPMI 1640 medium overnight at 100,000 x*g* at 4°C then filtering through 0.22μM pore size PVDF filters (Millex). Cells were washed twice in PBS and resuspended in the freshly prepared exosome-depleted medium at a concentration of 400,000 cells per mL. After 48 hours in culture, a volume of 120 mL of cells and culture supernatant were collected for each cell line and centrifuged at 2,000*g* for 10 minutes at 4°C. The cells pellet was washed twice with PBS then resuspended in FBS with 10% of DMSO and stored at -80°C.

### RNA extraction from exosomes and bone marrow selected cells

Total RNA was extracted from previously isolated exosomes using the miRNeasy Micro Kit (Qiagen) then concentrated with the RNA Clean & Concentrator-5 (Zymo). Primary WM cells were obtained from bone marrow biopsy samples using CD19+ microbeads selection (Miltenyi Biotec) as previously described and stored at -80°C[27]. Cells were homogenized with Qiashredder columns (Qiagen) and RNA was extracted from the homogenized lysate using the miRNeasy Micro Kit (Qiagen). The amount of RNA obtained was measured by fluorimetry with the Quant-iT RiboGreen RNA Assay Kit (Thermo Fisher Scientific).

### Analysis of exosomal microRNA expression

#### TaqMan assay

MiRNA profiling was performed using TaqMan Array Cards (Thermo Fisher Scientific). A total of 377 mature miRNAs based on miRbase v21 were interrogated on the A v2.1 card for each sample. Reverse transcription (RT) was performed using the TaqMan MicroRNA Reverse Transcription Kit and Megaplex RT Primers, Human Pool A v2.1 (Thermo Fisher Scientific) according to the manufacturer’s protocol, with a starting amount of RNA ranging from 3 to 25 ng. The RT products (4 μL) were pre-amplified in 20 μL reactions with the Megaplex PreAmp Primers Human Pool A v2.1 (10x) and the TaqMan PreAmp Master Mix (2x) (Thermo Fisher Scientific). The amplification was performed at 95°C for 10 min, 55°C for 2 min, 72°C for 2 min, then 16 cycles at 95°C for 15 seconds, 60°C for 4 min, followed by 99.9°C for 10 min, and 4°C on hold. Undiluted pre-amplification products (1x final concentration) were used to prepare the real-time qPCR samples with 2x TaqMan Master Mix II, no UNG (Thermo Fisher Scientific). All the TaqMan Array Cards were run on the Applied Biosystems 7900HT Fast Real-Time PCR System according to manufacturer’s protocol. All Ct values above 35 cycles were considered as undetectable. We normalized the data with a universal method for miRNA RT-qPCR data normalization using the mean expression value of all expressed miRNAs in a given sample[28].

#### Firefly multiplex circulating miRNA assay

To exclude any potential confounding effect due to the blood collection method (use of heparin or citrate EDTA-coated tubes) on the assay results, peripheral blood was collected from 6 patients in one tube containing heparin and one tube containing citrate EDTA at the same timepoint. Exosomes were isolated from the plasma of these matched samples as previously described. The Oncology panel of the Firefly Multiplex Circulating miRNA Assay (Abcam) was used to analyze these samples. Samples were scanned on a Guava 8HT flow cytometer (EMD Millipore). The signals measured by flow cytometry were converted into microRNA expression data by the Firefly Analysis Workspace software. The data was normalized with the geNORM algorithm which uses the signal of the three probes with the most stable expression across all samples[29]. The algorithm chose let-7i-5p, miR-103a-3p and miR-20a-5p as normalizer probes.

Furthermore, a custom panel comprised of 33 miRNAs (33-plex) was built to measure the expression levels of these miRNAs with the Firefly Multiplex Circulating miRNA Assay. The assay was performed in exosomes isolated from peripheral blood plasma and cell lines as previously described as well as in RNA from cell lines and bone marrow selected WM cells. Samples were scanned on a BD FACS CANTO II flow cytometer with a HTS unit (BD Biosciences). The signals measured by flow cytometry were converted into microRNA expression data by the Firefly Analysis Workspace software. The data was normalized using the geNORM algorithm[29]. The algorithm chose let-7i-5p, miR-20a-5p and miR-223-3p as normalizer probes.

### Statistical analysis

The primary outcome of interest was the expression of exosomal miRNA and how it correlates with disease stages in WM. Patients’ characteristics were presented as the median and interquartile range (IQR). Principal component analysis (PCA) was performed to distinguish the miRNA signatures between healthy donors and WM patients. The two-sample t-test was applied to explore the difference of each miRNA between two groups and Benjamini and Hochberg correction was used to attenuate cumulative type I errors in multiple comparisons[30]. Then we compared the mean of each miRNA expression between normal adults and WM patients in different disease status by using one-way analysis of variance (ANOVA). The post-hoc analyses tested the differences and linear trend between groups. All statistical analyses were performed in R (version 3.3.3) and SAS 9.2 software (SAS Institute Inc.).

## Results

To characterize disease progression from smoldering to symptomatic WM, we first isolated exosomes from plasma samples of healthy donors and patients with WM at progressive stages of the disease. The patient characteristics are included in Tables 1 and 2. Transmission electron microscopy with immunogold labeling for the exosome-specific markers CD63 and CD81 and NanoSight analysis confirmed the presence and the size of the exosomes in our samples (S1 Fig)[7].

**Table 1:** Clinical characteristics of Waldenström Macroglobulinemia patients–TaqMan Array Human MicroRNA A Cards.

	Total *n* = 16	Asymptomatic WM *n* = 9	Symptomatic WM *n* = 7	*P* value
Characteristics	*n* (%)	*n* (%)	*n* (%)	
Median age, years (IQR)	65 (56–70)	68 (56–70)	65 (56–66)	0.660
≥ 70	4 (25.0)	3 (33.3)	1 (14.3)	0.585
< 70	12 (75.0)	6 (66.7)	6 (85.7)	
Sex				
Male	10 (62.5)	6 (66.7)	4 (57.1)	1.000
Female	6 (37.5)	3 (33.3)	3 (42.9)	
Light chain				
Kappa	12 (75.0)	8 (88.9)	4 (57.1)	0.262
Lambda	4 (25.0)	1 (11.1)	3 (42.9)	
Family history for WM	2 (12.5)	1 (11.1)	1 (14.3)	1.000
Hemoglobin, g/dL				
Median (IQR)	12.4 (10.8–13.0)	13.0 (11.8–14.0)	11.8 (10.8–12.5)	0.078
Hemoglobin ≥ 11	10 (62.5)	6 (66.7)	4 (57.1)	0.559
β2-microglobulin				
Median (IQR)	2.3 (2.2–2.4)	2.2 (2.2–2.2)	2.4 (2.1–3.9)	0.467
≥ 3.0 mg/dL	1 (6.3)	0 (0.0)	1 (14.3)	1.000
M-spike	1.3 (0.8–2.1)	1.2 (0.9–1.6)	1.4 (0.7–2.5)	0.876
% bone marrow involvement	25 (18–50)	15 (15–20)	30 (30–90)	0.008
Deceased	0 (0.0)	0 (0.0)	0 (0.0)	

**Table 2:** Clinical characteristics of Waldenström Macroglobulinemia patients–Firefly Multiplex circulating miRNA assay.

	Total *n* = 62	Asymptomatic *n* = 25	Symptomatic naive *n* = 10	Relapsed *n* = 27	*P* value
Characteristics	*n* (%)	*n* (%)	*n* (%)	*n* (%)	
Median age, years (IQR)	68 (60–72)	69 (59–75)	71 (64–77)	66 (59–70)	0.307
≥ 70	25 (40.3)	12 (48.0)	5 (50.0)	8 (29.6)	0.319
< 70	37 (59.7)	13 (52.0)	5 (50.0)	19 (70.4)	
Sex					
Male	39 (62.9)	16 (64.0)	5 (50.0)	18 (66.7)	0.641
Female	23 (37.1)	9 (36.0)	5 (50.0)	9 (33.3)	
Light chain					
Kappa	44 (71.0)	17 (68.0)	9 (90.0)	18 (66.7)	0.349
Lambda	18 (29.0)	8 (32.0)	1 (10.0)	9 (33.3)	
Family history for WM	8 (12.9)	1 (4.0)	1 (10.0)	6 (22.2)	0.146
Hemoglobin, g/dL					
Median (IQR)	11.8 (10.8–13.6)	12.8 (11.8–14.2)	10.9 (10.4–12.4)	11.1 (10.0–12.0)	0.001
Hemoglobin ≥ 11	42 (67.7)	23 (92.0)	5 (50.0)	14 (51.9)	0.004
β2-microglobulin					
Median (IQR)	2.7 (2.1–4.1)	2.3 (1.9–2.7)	4.2 (2.4–4.8)	3.2 (2.4–4.1)	0.003
≥ 3.0 mg/dL	22 (35.5)	3 (12.0)	5 (50.0)	14 (51.9)	0.011
IgM	2390 (1287–3400)	1937 (1018–2521)	2065 (1130–2740)	3130 (2226–4490)	0.006
M-spike	1.3 (0.9–1.9)	1.1 (0.9–1.4)	1.3 (0.9–1.9)	1.8 (0.9–2.9)	0.121
% bone marrow involvement	40 (20–70)	30 (20–50)	45 (20–70)	55 (28–80)	0.128
Deceased	8 (12.9)	1 (4.0)	0 (0.0)	7 (25.9)	

IQR, interquartile range

**Fig 1:**
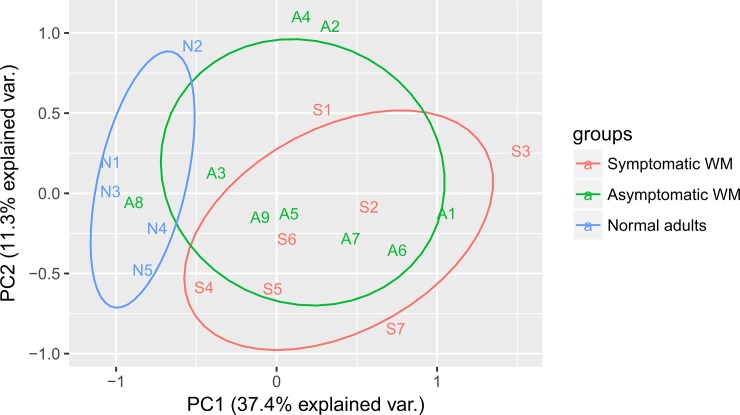
Global exosomal miRNA expression in WM patient groups. Principle Component analysis of TaqMan Array Cards Results representing the variation of exosomal miRNAs’ expression in samples from healthy controls (N1-N5), patients with asymptomatic WM (A1-A9) and patients with symptomatic naïve WM (S1-S7).

We next used the TaqMan Array Human MicroRNA A Cards to identify miRNAs differentially expressed in the circulating exosomes of healthy controls (HC, n = 5) and patients with smoldering WM (AWM, n = 9) or previously untreated symptomatic WM (SWM, n = 7). This assay enables the detection of 377 human miRNAs by qRT-PCR. We found 47 microRNAs with significantly different levels of expression between the groups (ANOVA test with Benjamini-Hochberg correction, *p* < 0.05) (S1 Table). The Principle Component Analysis of the assay results showed that patients with SWM can be distinguished from HC based on their exosomal miRNA levels. In contrast, the miRNA expression profiles of patients with AWM partially overlapped with those of both the HC and SWM groups, indicative of the heterogeneity of this group of MGUS/smoldering WM that is not well defined (Fig 1). Importantly, we observed no correlation between the bone marrow involvement of patients with AWM and the similarity of these patient’s microRNA signatures to either the HC group or the SWM group (S2 Table). This suggests that the differences observed are not merely due to a higher number of tumor derived-exosomes in the peripheral blood resulting from a higher BM involvement.

Of note, a number of the plasma samples available for our study were collected in tubes containing heparin, which is known to inhibit PCR amplification. [31] Therefore we chose for further analysis the Firefly Multiplex Circulating miRNA Assay which is not affected by the use of heparin. To validate our choice, we compared the microRNA levels measured by this assay in exosomes isolated from peripheral blood collected from six patients with WM in both heparin and citrate EDTA-coated tubes. There was no difference in the level of miRNAs observed between matching samples (S2 Fig).

We therefore designed a custom panel to measure with this assay the expression of 33 miRNAs that were selected based on the TaqMan assay results and on a review of the literature for microRNAs with a role in WM pathogenesis [32–36] (S3 Table). The 33-plex assay was used to analyze the circulating exosomes of 70 patient samples including 8 HC, 25 patients with AWM and 37 patients with SWM (including previously untreated WM (n = 10), relapsed WM (n = 22) and refractory WM (n = 5)).

**Fig 2:**
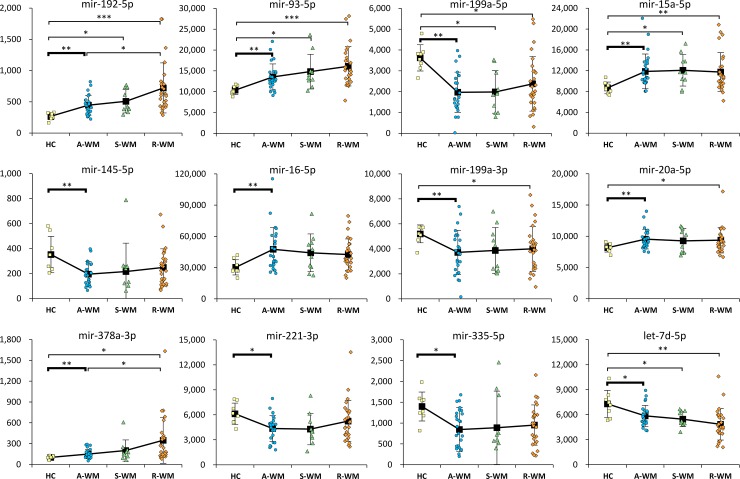
Exosomal miRNAs differentially expressed in patients with A-WM compared to healthy controls. Distribution (Mean + SEM) of microRNA expression in Healthy Controls (HC) or WM patients at progressive stages of disease (A-WM = Asymptomatic WM, S-WM = Symptomatic naive WM and R-WM = Relapsed WM) measured by the Abcam Firefly Multiplex circulating miRNA assay (33-plex) for miRNAs with a statistically significant difference of expression between the A-WM and HC groups. *P*-values were determined by a two-sample t-test with Benjamini-Hochberg correction applied for False Discovery Rate adjustment.

A two-sample t-test between successive groups of disease progression revealed 12 microRNAs differentially expressed between the AWM and HC groups (two-sample t-test with Benjamini-Hochberg correction; adjusted *p* < 0.05). Interestingly, none of the microRNAs of the panel showed a significant difference of expression between the AWM and the SWM groups and between the SWM and relapsed WM groups. (Fig 2 and S4 Table)

**Fig 3:**
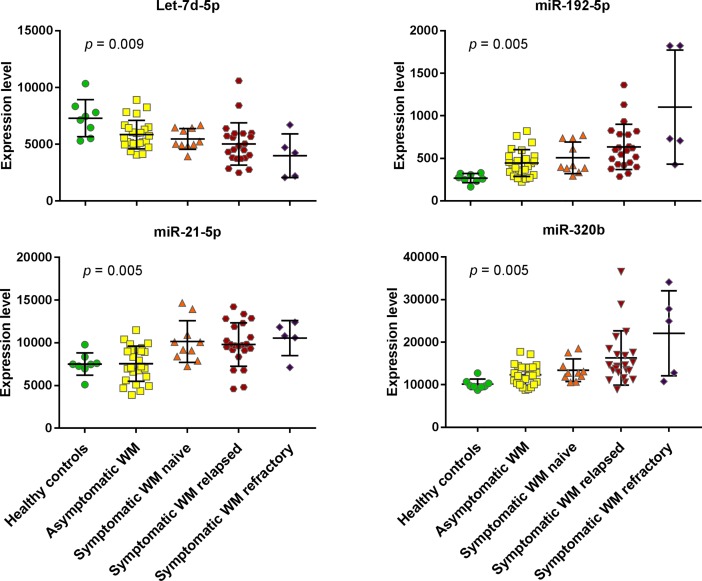
Exosomal miRNAs correlating with WM progression. A) Distribution (Mean + SEM) of microRNA expression in Healthy Controls or WM patients at progressive stages of disease measured by the Abcam Firefly Multiplex circulating miRNA assay (33-plex). Significance was assessed by a one-way ANOVA (*p* < 0.01) and a test for linear trend (*p-*for-trend < 0.005) with Benjamini-Hochberg correction applied for False Discovery Rate adjustment.

Moreover, a one-way ANOVA with a test for linear trend was performed on the results of the 33-plex assay. This analysis revealed a group of 4 miRNAs with a statistically significant linear trend between their expression levels in circulating exosomes and the stage of WM progression (ANOVA *p* < 0.01 and *p-*for-trend < 0.005 adjusted with Benjamini-Hochberg correction). Three of these miRNAs, namely miR-192-5p, miR-21-5p, and mir-320b showed increased expression levels with disease progression while the expression of the remaining one, let-7d, decreased with disease stage (Fig 3 and S4 Table).

**Fig 4:**
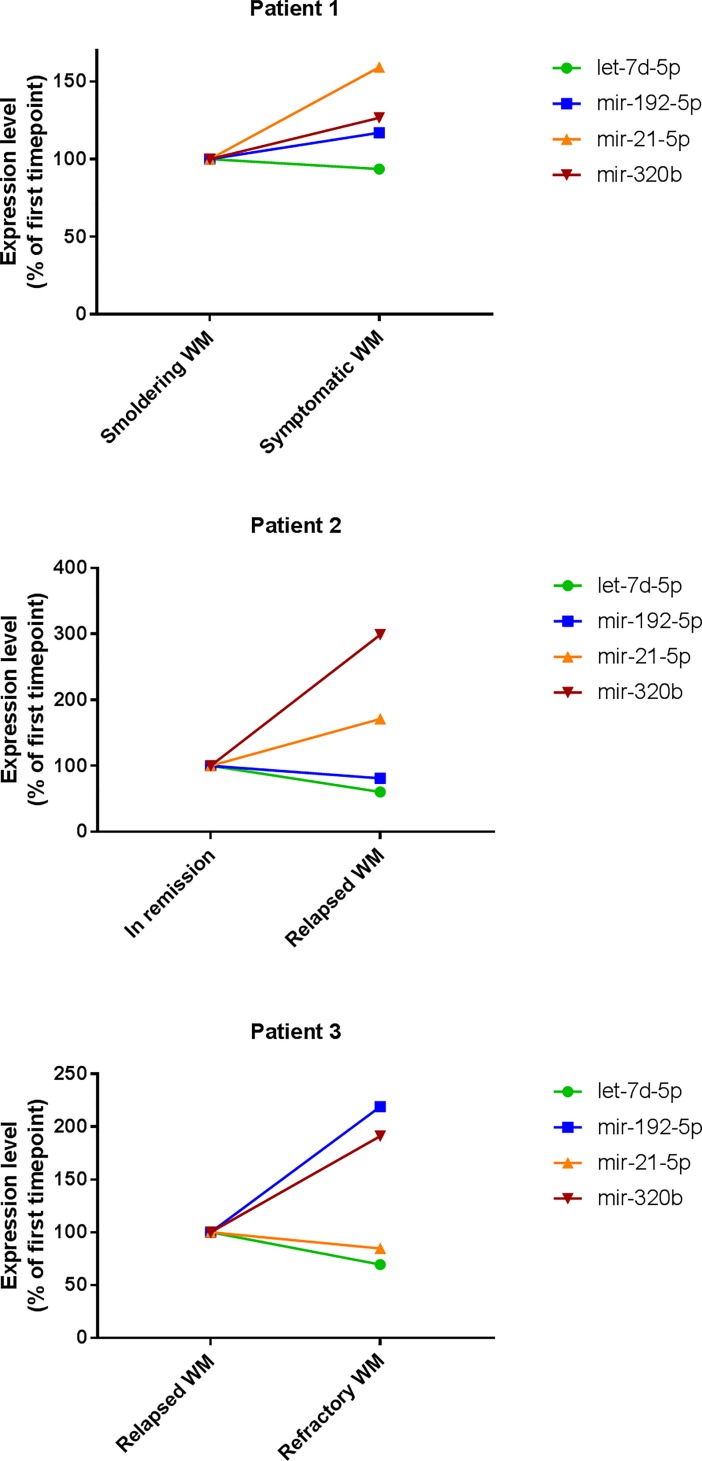
Assessment of exosomal miRNAs with diagnostic potential in sequential WM patients’ samples. MicroRNA expression levels measured by the Abcam Firefly Multiplex circulating miRNA assay (33-plex) in sequential samples from patients with WM during distinct phases of disease progression. For each microRNA, the expression level of the second timepoint is represented as a percentage of the expression level of the first timepoint.

Additionally, we analyzed sequential plasma samples at different stages of disease progression for three of our cohort’s patients with the 33-plex assay. The levels of miR-21-5p and miR-192-5p increased with disease progression in 2 out of the 3 patients while a decrease of let-7d and an increase of miR-320b were observed in all 3 patients, consistently with our findings (Fig 4).

Moreover, we sought to assess whether the miRNA content of respectively tumor-derived exosomes and circulating exosomes reflects the miRNA expression profiles of tumor cells. We examined the microRNA expression levels of CD19-selected bone marrow tumor cells and circulating exosomes from matching peripheral blood plasma samples in 6 patients with WM. Samples were analyzed with the 33-plex assay and the mean Pearson correlation coefficient between the exosomal and cellular miRNA levels detected in the six patients’ samples was 0.52 (S3A Fig). Moreover, in two WM cell lines BCWM.1 and MWCL-1, we compared the miRNA expression levels between the cells and the exosomes derived from these cells.[37, 38] The Pearson correlation coefficient between the miRNA levels of the cellular and exosomal fractions was of 0.81 for BCWM.1 cells and 0.90 for MWCL-1 cells (S3B Fig).

## Discussion

Exosomes have been shown to mediate many aspects of tumor development and progression such as immune evasion, metastasis and drug resistance and to enable cross-talk between tumor cells and their microenvironment[7–9, 39, 40]. The effect of exosomes on tumorigenesis can occur through their miRNA content[10–14].

Circulating exosomes can be isolated from the peripheral blood of patients, making them an attractive mean to identify molecular markers of disease progression. In our study, we analyzed the microRNA content of exosomes from the plasma of patients at different stages of WM as well as healthy controls for comparison. Our results show that patients with WM can be distinguished from healthy controls based on the microRNA expression levels in their exosomes. Interestingly, patients with asymptomatic WM displayed expression levels of exosomal miRNAs comparable to those of symptomatic untreated patients, indicating that changes in the microRNA content of circulating exosomes occur before WM progresses into a symptomatic state. These intriguing findings suggest that the bone marrow and immune environments might evolve early after WM initiation and even potentially at the MGUS stage. This hypothesis is also supported by the high frequency of the MYD88 L265P mutation observed in patients with IgM MGUS and AWM. [41, 42] It might also indicate that treatment of WM at the asymptomatic stage could be an interesting therapeutic strategy to explore.

Although the number of samples with asymptomatic WM used in this study was small, future larger studies may help define those that may have a miRNA signature that is “normal or MGUS-like” vs. those that show a “symptomatic WM signature” and are likely to progress to symptomatic disease. Potentially, these patients may benefit from early therapeutic interventions before the development of clinically evident end-organ damage.

Most significantly, we identified a group of four miRNAs whose levels of expression correlated significantly with stages of disease progression (ANOVA *p* < 0.01 and *p*- for trend < 0.05). The levels of expression of miR-192-5p, miR-320b and miR-21-5p increased with the disease stage whereas the expression of let-7d was downregulated. In addition, sequential samples from 3 patients showed that indeed the level of expression of these miRNAs changes significantly with disease progression, indicating that these 4 miRNA markers could be used to track disease progression. Further studies in larger cohorts are required to confirm this observation.

Interestingly, let-7d and miR-21-5p have defined roles in cancer as a tumor suppressor miRNA and oncogenic miRNA respectively, supporting their biological relevance in our study. The let-7 family of miRNAs are well-known tumor suppressors in several cancer types including multiple myeloma, another plasma cell malignancy[43–45]. A recent study by our group identified low levels of let-7b in circulating exosomes as a marker of multiple myeloma progression[25]. Let-7 exerts its tumor suppressor activity by regulating different oncogenes including MYC, RAS and CCND1[46]. Moreover, it is regulated by the RNA-binding protein LIN28 which had shown aberrant expression in cancer[46]. Conversely, miR-21-5p is recognized as a ubiquitous oncogene and is upregulated in most solid tumors and a number of hematological malignancies[47]. miR-21 exerts its activity by regulating numerous aspects of the tumorigenesis process, notably through the regulation of numerous tumor suppressor genes including PTEN, PDCD4 and CDKAP1[47]. Importantly, overexpression of miR-21 has been shown to promote a pre-B malignant lymphoid-like phenotype in mice[48].

Both tumor promoting and inhibiting effects have been described for miR-192-5p depending on the type of cancer studied[49–53]. The specific role of this miRNA remains to be elucidated in the specific context of WM. In the same way, studies have reported an anti-tumor activity for miR-320b, however it has also been found as upregulated in the peripheral blood of WM patients compared to patients with IgM MGUS or IgM multiple myeloma and healthy donors[36, 54–56]. Moreover, it has been shown that members of the miR-320 family are preferentially sorted into exosomes derived from both healthy and malignant cells[21].

A study similar to ours was conducted in a cohort of patients with Multiple Myeloma revealed a different set of microRNAs with a prognostic value in this disease[25]. However, one of these miRNAs was let-7b which belongs to the same family of microRNAs than let-7d. Furthermore, another recent study suggests a potential prognostic role for miR-21 serum levels in Multiple Myeloma[57].

An interesting feature of circulating exosomes is that the study of their content can provide information not only on tumor cells but also on tumor-supportive tissues across the body. Our results suggest that exosomes secreted by tumor cells have similar miRNA expression profiles to the cells they originate from. As expected, we observed a lower correlation between the miRNA expression levels in tumor cells and circulating exosomes possibly due to the fact that peripheral blood plasma contains exosomes derived from various cell types including tumor cells, microenvironment cells and immune cells. Nevertheless, the differences in miRNA expression signatures observed in circulating exosomes at different disease states can provide valuable information on changes occurring during disease progression in tumor supportive tissues and the immune system.

In conclusion, we profiled exosomes from the peripheral blood of patients with WM at different stages of disease progression. To our knowledge, this is the first study to define the miRNA exosomal content of WM patient samples. Together, our results suggest that changes in the microRNA content of circulating exosomes occur at early stages of disease progression before symptoms appear in patients. This might indicate that changes in tumor cells and their microenvironment occur before WM progresses into a symptomatic state.

Moreover, we identified a small group of miRNAs whose expression correlated with the disease status of patients, notably the known tumor suppressor miRNA let-7d and the oncogene miR-21 as well as miR-192 and miR-320b. Further evaluation of these miRNAs’ specific effect in WM cells could help us gain further insights on the mechanisms underlying WM pathogenesis and reveal their potential role as novel therapeutic targets for this disease.

## Supporting information

S1 Fig Characterization of exosomes isolated from peripheral blood plasma samples. A) Ultracentrifugation method used for isolation of exosomes from peripheral blood plasma samples and cell culture supernatant. B) Imaging of exosomes isolated from peripheral blood plasma by electron microscopy imaging after staining with human anti-CD63 and anti-CD81 antibodies (magnification 30,000x). C) Analysis of particle size in exosomes isolated from peripheral blood plasma of a patient with WM (sample diluted 1:100 in PBS) with a NanoSight NS300 Instrument (Malvern).
(PDF)

S2 Fig Effect of blood drawing methods on exosomal miRNA expression. Correlation of microRNA expression levels measured by the Abcam Firefly Multiplex circulating miRNA assay (33-plex) in exosomes isolated from matching peripheral blood plasma samples collected in tubes containing citrate EDTA or heparin in 6 patients with WM.
(TIF)

S3 Fig Correlation of cellular and exosomal microRNA expression. miRNA expression levels were measured with the Firefly Multiplex Circulating miRNA assay (Abcam) in A) patients with relapsed symptomatic WM (CD19-selected bone marrow cells vs. circulated exosomes) and B) WM cell lines (cells vs. cell-derived exosomes).
(TIF)

S1 Table Significant exosomal miRNAs measured by the TaqMan Array Cards. ANOVA test results (with Benjamini-Hochberg correction) and *p*-values and *p*- for trend after Benjamini and Hochberg correction are shown for exosomal miRNA expression levels measured with the TaqMan Array Cards (Human Pool A v2.1).
(PDF)

S2 Table Bone marrow involvement of patients included in the TaqMan Array analysis. Percentage of bone marrow (BM) infiltration by tumor cells in bone marrow biopsy samples from patients included in the analysis of circulating exosome miRNA expression with the TaqMan Array Cards (Human Pool A v2.1). (N/A: bone marrow infiltration percentage not available)
(PDF)

S3 Table microRNA panel of the 33-plex Firefly Multiplex circulating miRNA assay. (DOCX)

S4 Table 33-plex Firefly Multiplex circulating miRNA assay results. An ANOVA test and two-samples t-test between 2 groups (Asymptomatic WM vs. Healthy controls and Symptomatic naïve WM vs. Asymptomatic WM) were conducted and *p*-values and *p*- for trend after Benjamini and Hochberg correction are shown.
(PDF)

S1 File Results of the TaqMan Array analysis. Expression levels of microRNAs measured in circulating exosomes samples from patients with WM and healthy controls by the TaqMan Array Cards (Human Pool A v2.1) and after normalization using the mean expression value of all expressed miRNAs in a given sample.
(XLSX)

S2 File Results of the Firefly 33-plex assay. Expression levels of microRNAs measured in circulating exosomes samples from patients with WM and healthy controls by the Abcam Firefly Multiplex circulating miRNA assay (33-plex) and after normalization with the geNORM algorithm using the expression levels of the 3 most stable probes among samples (hsa-let-7i; hsa-miR-20a-5p and hsa-miR-223-3p).
(XLSX)
