# Supplementary figures and images for: Profiling of circulating exosomal miRNAs in patients with Waldenström Macroglobulinemia
Source: PLoS One. 2018 Oct 4;13(10):e0204589. doi: 10.1371/journal.pone.0204589 (PMC6171840; doi:10.1371/journal.pone.0204589)

A)

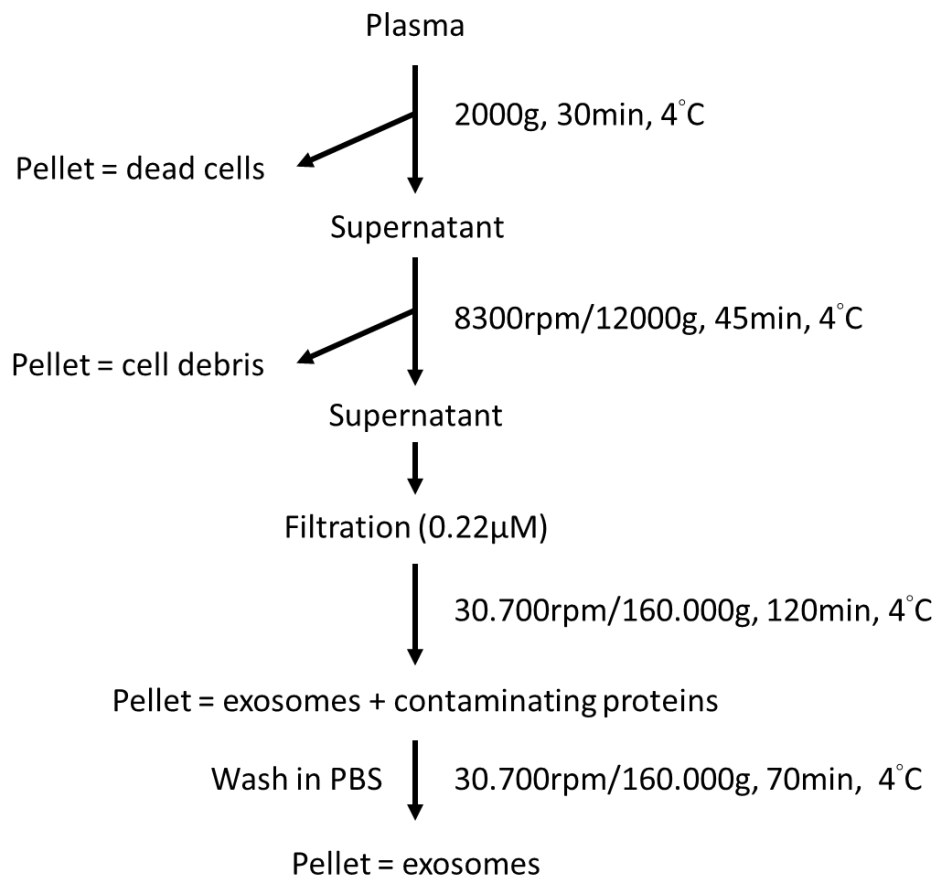

B)

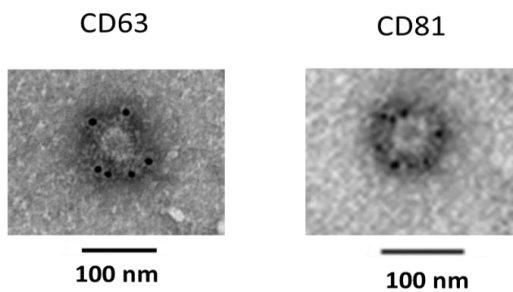

C)

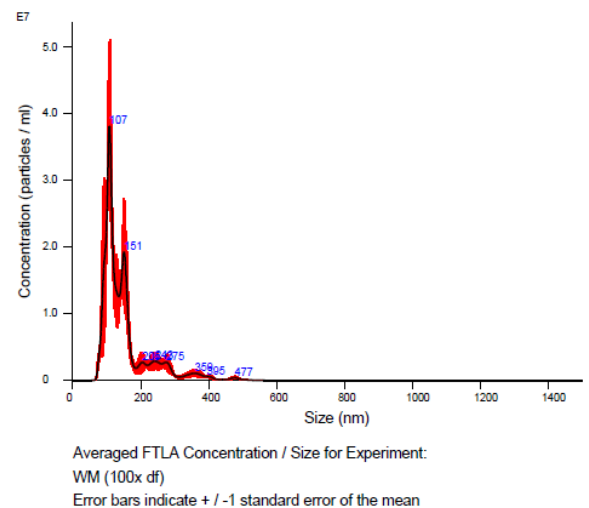

Supplement: S1 Fig — A) Ultracentrifugation method used for isolation of exosomes from peripheral blood plasma samples and cell culture supernatant. B) Imaging of exosomes isolated from peripheral blood plasma by electron microscopy imaging after staining with human anti-CD63 and anti-CD81 antibodies (magnification 30,000x). C) Analysis of particle size in exosomes isolated from peripheral blood plasma of a patient with WM (sample diluted 1:100 in PBS) with a NanoSight NS300 Instrument (Malvern). (PDF) [file pone.0204589.s001.pdf]

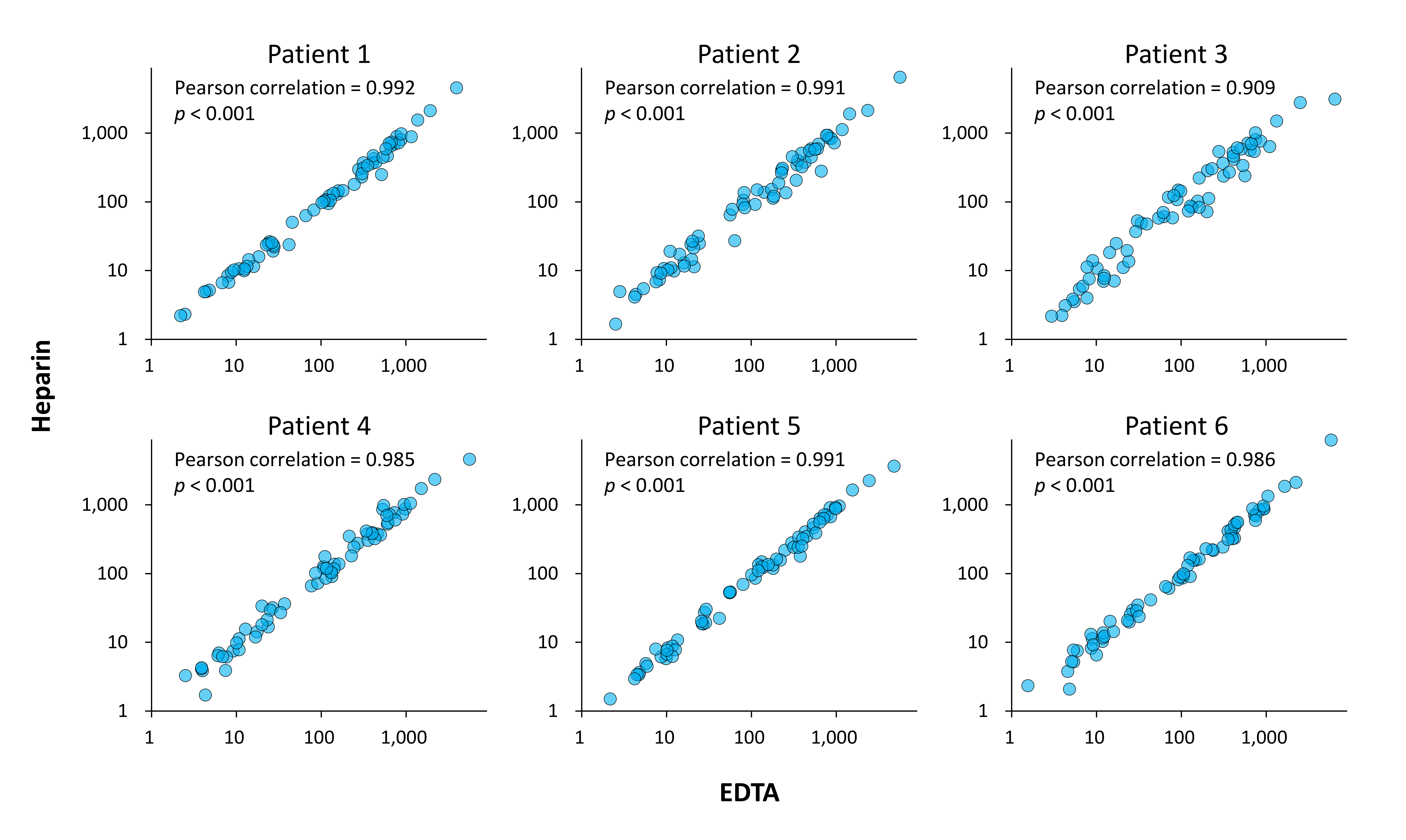

Supplement: S2 Fig — Correlation of microRNA expression levels measured by the Abcam Firefly Multiplex circulating miRNA assay (33-plex) in exosomes isolated from matching peripheral blood plasma samples collected in tubes containing citrate EDTA or heparin in 6 patients with WM. (TIF) [file pone.0204589.s002.tif]

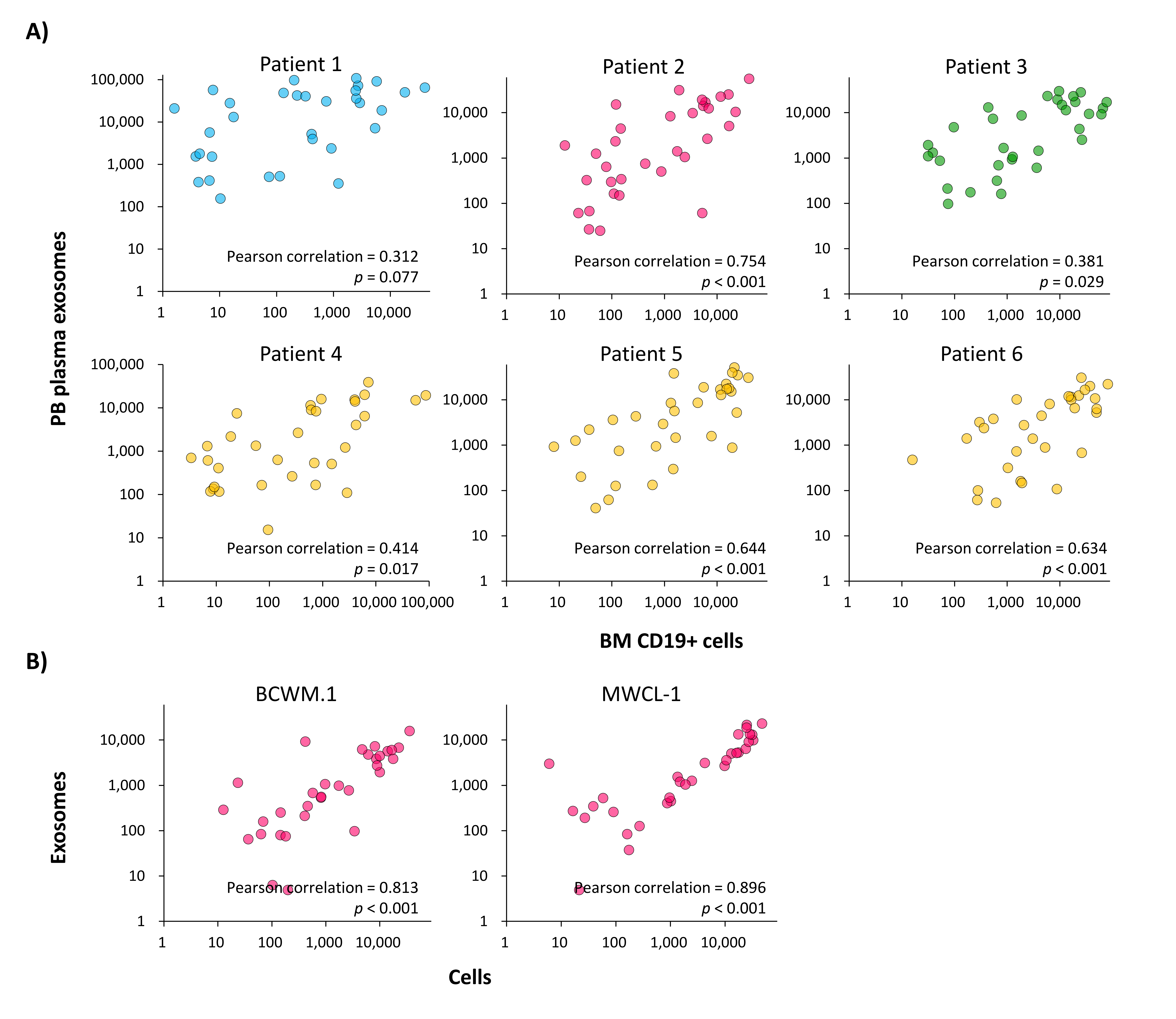

Supplement: S3 Fig — miRNA expression levels were measured with the Firefly Multiplex Circulating miRNA assay (Abcam) in A) patients with relapsed symptomatic WM (CD19-selected bone marrow cells vs. circulated exosomes) and B) WM cell lines (cells vs. cell-derived exosomes). (TIF) [file pone.0204589.s003.tif]
