# Supplementary material for: Profiling of circulating exosomal miRNAs in patients with Waldenström Macroglobulinemia
Source: PLoS One. 2018 Oct 4;13(10):e0204589. doi: 10.1371/journal.pone.0204589 (PMC6171840; doi:10.1371/journal.pone.0204589)
Supplement: S1 Table — ANOVA test results (with Benjamini-Hochberg correction) and p-values and p- for trend after Benjamini and Hochberg correction are shown for exosomal miRNA expression levels measured with the TaqMan Array Cards (Human Pool A v2.1). (PDF) [file pone.0204589.s004.pdf]

microRNA – TaqMan probe number	*p*-value (ANOVA)	*p*- for trend
hsa-miR-145-002278	0.012	0.013
hsa-miR-28-000411	0.012	0.011
hsa-miR-501-3p-002435	0.012	0.011
hsa-miR-26a-000405	0.014	0.011
hsa-miR-27b-000409	0.014	0.013
hsa-miR-744-002324	0.014	0.011
hsa-miR-181a-000480	0.015	0.011
hsa-miR-200b-002251	0.015	0.020
hsa-miR-23b-000400	0.015	0.011
hsa-miR-24-000402	0.015	0.013
hsa-miR-320-002277	0.015	0.011
hsa-miR-491-3p-002360	0.015	0.013
hsa-miR-500-002428	0.015	0.013
hsa-miR-519d-002403	0.015	0.062
hsa-miR-548a-5p-002412	0.015	0.011
hsa-miR-518b-001156	0.015	0.017
hsa-miR-221-000524	0.017	0.013
hsa-miR-103-000439	0.017	0.013
hsa-miR-423-5p-002340	0.018	0.013
hsa-miR-335-000546	0.021	0.015
hsa-miR-130a-000454	0.023	0.015
hsa-miR-130b-000456	0.023	0.035
hsa-miR-139-3p-002313	0.023	0.039
hsa-miR-517b-001152	0.023	0.013
hsa-miR-518a-3p-002397	0.023	0.013
hsa-miR-518d-5p-002389	0.023	0.013
hsa-miR-519a-002415	0.023	0.016
hsa-miR-519e-002370	0.023	0.014
hsa-miR-501-001047	0.024	0.011
hsa-miR-515-5p-001112	0.024	0.022
hsa-miR-891a-002191	0.024	0.013
hsa-miR-574-3p-002349	0.027	0.014
hsa-miR-98-000577	0.027	0.016
hsa-miR-223-002295	0.028	0.039
hsa-let-7c-000379	0.029	0.013
hsa-let-7d-002283	0.029	0.015
hsa-miR-181c-000482	0.029	0.062
hsa-miR-191-002299	0.029	0.021
hsa-miR-518d-001159	0.029	0.158
hsa-miR-520e-001119	0.029	0.074
hsa-miR-30b-000602	0.030	0.025
hsa-miR-199a-3p-002304	0.032	0.021
hsa-miR-542-5p-002240	0.033	0.013
hsa-miR-193b-002367	0.041	0.021
hsa-miR-328-000543	0.042	0.041
hsa-miR-326-000542	0.043	0.070
hsa-miR-503-001048	0.043	0.074
