# Supplementary material for: Profiling of circulating exosomal miRNAs in patients with Waldenström Macroglobulinemia
Source: PLoS One. 2018 Oct 4;13(10):e0204589. doi: 10.1371/journal.pone.0204589 (PMC6171840; doi:10.1371/journal.pone.0204589)
Supplement: S2 Table — Percentage of bone marrow (BM) infiltration by tumor cells in bone marrow biopsy samples from patients included in the analysis of circulating exosome miRNA expression with the TaqMan Array Cards (Human Pool A v2.1). (N/A: bone marrow infiltration percentage not available) (PDF) [file pone.0204589.s005.pdf]

Patient		BM infiltration (%)
Asymptomatic WM	A1	15
	A2	9
	A3	N/A
	A4	N/A
	A5	20
	A6	15
	A7	N/A
	A8	20
	A9	N/A
Symptomatic naïve WM	S1	90
	S2	90
	S3	30
	S4	30
	S5	70
	S6	20
	S7	30
