# Supplementary material for: Profiling of circulating exosomal miRNAs in patients with Waldenström Macroglobulinemia
Source: PLoS One. 2018 Oct 4;13(10):e0204589. doi: 10.1371/journal.pone.0204589 (PMC6171840; doi:10.1371/journal.pone.0204589)
Supplement: S3 Table — (DOCX) [file pone.0204589.s006.docx]

| Firefly Multiplex circulating assay – 33-plex panel | Reason for inclusion in the panel |
| --- | --- |
| hsa-let-7d-5p | Litterature [1, 2] |
| hsa-let-7i-5p | Normalizer miRNA |
| hsa-mir-103a-3p | Normalizer miRNA |
| hsa-mir-107 | Taqman assay |
| hsa-mir-10b-5p | Taqman assay + literature [3, 4] |
| hsa-mir-130a-3p | Taqman assay |
| hsa-mir-139-3p | Taqman assay |
| hsa-mir-139-5p | Taqman assay |
| hsa-mir-145-5p | Taqman assay + literature [5] |
| hsa-mir-155-5p | Literature [6-8] |
| hsa-mir-15a-5p | Literature [9] |
| hsa-mir-16-1-3p | Literature [9] |
| hsa-mir-16-5p | Hemolysis marker |
| hsa-mir-181a-5p | Taqman assay |
| hsa-mir-181b-5p | Same cluster as miR-181a + literature [10, 11] |
| hsa-mir-192-5p | Taqman assay |
| hsa-mir-199a-3p | Taqman assay |
| hsa-mir-199a-5p | Taqman assay |
| hsa-mir-20a-5p | Normalizer miRNA |
| hsa-mir-21-5p | Taqman assay + literature [12] |
| hsa-mir-221-3p | Taqman assay |
| hsa-mir-222-3p | Taqman assay |
| hsa-mir-223-3p | Taqman assay |
| hsa-mir-27b-3p | Taqman assay |
| hsa-mir-320a | Taqman assay + literature [13] |
| hsa-mir-320b | Same cluster as mir-320b + literature [13] |
| hsa-mir-324-5p | Taqman assay |
| hsa-mir-335-5p | Taqman assay |
| hsa-mir-339-3p | Taqman assay |
| hsa-mir-339-5p | Taqman assay |
| hsa-mir-378a-3p | Literature [14-16] |
| hsa-mir-500a-5p | Taqman assay |
| hsa-mir-93-5p | Literature [10] |

1. Wang T, Wang G, Hao D, Liu X, Wang D, Ning N, et al. Aberrant regulation of the LIN28A/LIN28B and let-7 loop in human malignant tumors and its effects on the hallmarks of cancer. Mol Cancer. 2015;14:125. doi: 10.1186/s12943-015-0402-5. PubMed PMID: 26123544; PubMed Central PMCID: PMCPMC4512107.

2. Manier S, Powers JT, Sacco A, Glavey SV, Huynh D, Reagan MR, et al. The LIN28B/let-7 axis is a novel therapeutic pathway in multiple myeloma. Leukemia. 2017;31(4):853-60. doi: 10.1038/leu.2016.296. PubMed PMID: 27773931; PubMed Central PMCID: PMCPMC5382134.

3. Ma L. Role of miR-10b in breast cancer metastasis. Breast Cancer Res. 2010;12(5):210. Epub 2010/11/12. doi: 10.1186/bcr2720. PubMed PMID: 21067538; PubMed Central PMCID: PMCPMC3096969.

4. Singh R, Pochampally R, Watabe K, Lu Z, Mo YY. Exosome-mediated transfer of miR-10b promotes cell invasion in breast cancer. Mol Cancer. 2014;13:256. Epub 2014/11/28. doi: 10.1186/1476-4598-13-256. PubMed PMID: 25428807; PubMed Central PMCID: PMCPMC4258287.

5. Boominathan L. The guardians of the genome (p53, TA-p73, and TA-p63) are regulators of tumor suppressor miRNAs network. Cancer Metastasis Rev. 2010;29(4):613-39. Epub 2010/10/06. doi: 10.1007/s10555-010-9257-9. PubMed PMID: 20922462.

6. Sacco A, Ghobrial IM, Roccaro AM. Epigenetics in Waldenstrom's macroglobulinemia. Epigenomics. 2010;2(5):691-6. Epub 2011/11/30. doi: 10.2217/epi.10.42. PubMed PMID: 22122052.

7. Zhang Y, Roccaro AM, Rombaoa C, Flores L, Obad S, Fernandes SM, et al. LNA-mediated anti-miR-155 silencing in low-grade B-cell lymphomas. Blood. 2012;120(8):1678-86. Epub 2012/07/17. doi: 10.1182/blood-2012-02-410647. PubMed PMID: 22797699.

8. Gaudette BT, Dwivedi B, Chitta KS, Poulain S, Powell D, Vertino P, et al. Low expression of pro-apoptotic Bcl-2 family proteins sets the apoptotic threshold in Waldenstrom macroglobulinemia. Oncogene. 2016;35(4):479-90. Epub 2015/04/22. doi: 10.1038/onc.2015.103. PubMed PMID: 25893290; PubMed Central PMCID: PMCPMC4874246.

9. Aqeilan RI, Calin GA, Croce CM. miR-15a and miR-16-1 in cancer: discovery, function and future perspectives. Cell Death Differ. 2010;17(2):215-20. Epub 2009/06/06. doi: 10.1038/cdd.2009.69. PubMed PMID: 19498445.

10. Yu SC, Chen SU, Lu W, Liu TY, Lin CW. Expression of CD19 and lack of miR-223 distinguish extramedullary plasmacytoma from multiple myeloma. Histopathology. 2011;58(6):896-905. Epub 2011/03/16. doi: 10.1111/j.1365-2559.2011.03793.x. PubMed PMID: 21401705.

11. Lionetti M, Musto P, Di Martino MT, Fabris S, Agnelli L, Todoerti K, et al. Biological and clinical relevance of miRNA expression signatures in primary plasma cell leukemia. Clin Cancer Res. 2013;19(12):3130-42. Epub 2013/04/25. doi: 10.1158/1078-0432.CCR-12-2043. PubMed PMID: 23613318.

12. Pfeffer SR, Yang CH, Pfeffer LM. The Role of miR-21 in Cancer. Drug Dev Res. 2015;76(6):270-7. Epub 2015/06/18. doi: 10.1002/ddr.21257. PubMed PMID: 26082192.

13. Kubiczkova Besse L, Sedlarikova L, Kryukov F, Nekvindova J, Radova L, Almasi M, et al. Combination of serum microRNA-320a and microRNA-320b as a marker for Waldenstrom macroglobulinemia. Am J Hematol. 2015;90(3):E51-2. Epub 2014/11/28. doi: 10.1002/ajh.23910. PubMed PMID: 25428891.

14. Lee DY, Deng Z, Wang CH, Yang BB. MicroRNA-378 promotes cell survival, tumor growth, and angiogenesis by targeting SuFu and Fus-1 expression. Proc Natl Acad Sci U S A. 2007;104(51):20350-5. Epub 2007/12/14. doi: 10.1073/pnas.0706901104. PubMed PMID: 18077375; PubMed Central PMCID: PMCPMC2154434.

15. Yuan Y, Kluiver J, Koerts J, de Jong D, Rutgers B, Abdul Razak FR, et al. miR-24-3p Is Overexpressed in Hodgkin Lymphoma and Protects Hodgkin and Reed-Sternberg Cells from Apoptosis. The American Journal of Pathology. 2017;187(6):1343-55. doi: 10.1016/j.ajpath.2017.02.016.

16. Ho CS, Noor SM, Nagoor NH. MiR-378 and MiR-1827 Regulate Tumor Invasion, Migration and Angiogenesis in Human Lung Adenocarcinoma by Targeting RBX1 and CRKL, Respectively. J Cancer. 2018;9(2):331-45. Epub 2018/01/19. doi: 10.7150/jca.18188. PubMed PMID: 29344280; PubMed Central PMCID: PMCPMC5771341.
