# Supplementary material for: Profiling of circulating exosomal miRNAs in patients with Waldenström Macroglobulinemia
Source: PLoS One. 2018 Oct 4;13(10):e0204589. doi: 10.1371/journal.pone.0204589 (PMC6171840; doi:10.1371/journal.pone.0204589)
Supplement: S4 Table — An ANOVA test and two-samples t-test between 2 groups (Asymptomatic WM vs. Healthy controls and Symptomatic naïve WM vs. Asymptomatic WM) were conducted and p-values and p- for trend after Benjamini and Hochberg correction are shown. (PDF) [file pone.0204589.s007.pdf]

	ANOVA		Two-sample t-test	
			Asymptomatic WM vs. Healthy controls	Symptomatic naïve WM vs. asymptomatic WM
microRNA	*p* value	*p* for trend	*p* value	*p* value
hsa_mir_192_5p	0.005	0.003	0.001	0.771
hsa_let_7d_5p	0.009	0.003	0.036	0.841
hsa_mir_93_5p	0.014	0.003	0.001	0.771
hsa_mir_320b	0.005	0.004	0.053	0.771
hsa_mir_21_5p	0.005	0.005	0.977	0.771
hsa_mir_320a	0.006	0.007	0.080	0.984
hsa_mir_378a_3p	0.018	0.028	0.017	0.771
hsa_mir_103a_3p	0.114	0.028	0.329	0.771
hsa_mir_199a_5p	0.016	0.034	0.001	0.992
hsa_mir_139_5p	0.179	0.046	0.491	0.771
hsa_mir_16_1_3p	0.008	0.050	0.502	0.992
hsa_mir_15a_5p	0.191	0.076	0.002	0.771
hsa_mir_181a_5p	0.163	0.122	0.245	0.992
hsa_mir_222_3p	0.126	0.147	0.825	0.771
hsa_mir_335_5p	0.198	0.158	0.028	0.992
hsa_mir_500a_5p	0.248	0.158	0.245	0.771
hsa_mir_145_5p	0.179	0.214	0.009	0.992
hsa_mir_16_5p	0.198	0.214	0.009	0.992
hsa_mir_199a_3p	0.300	0.214	0.009	0.992
hsa_mir_27b_3p	0.322	0.214	0.088	0.106
hsa_mir_20a_5p	0.396	0.228	0.009	0.992
hsa_mir_339_5p	0.230	0.234	0.245	0.771
hsa_mir_181b_5p	0.179	0.367	0.704	0.992
hsa_mir_107	0.496	0.367	0.866	0.992
hsa_mir_221_3p	0.179	0.377	0.021	0.771
hsa_mir_339_3p	0.496	0.485	0.664	0.771
hsa_mir_223_3p	0.742	0.485	0.264	0.992
hsa_mir_139_3p	0.725	0.547	0.183	0.992
hsa_mir_130a_3p	0.288	0.733	0.059	0.771
hsa_mir_324_5p	0.496	0.733	0.245	0.992
hsa_mir_10b_5p	0.496	0.853	0.115	0.771
hsa_mir_155_5p	0.833	0.875	0.977	0.992
hsa_let_7i_5p	0.872	0.910	0.596	0.771
